# A Practical Approach Based on Analytic Deformable Algorithm for Scenic Image Registration

**DOI:** 10.1371/journal.pone.0066656

**Published:** 2013-06-21

**Authors:** Wei-Yen Hsu

**Affiliations:** Department of Information Management, National Chung Cheng University, Chiayi County, Taiwan; University of Adelaide, Australia

## Abstract

**Background:**

Image registration is to produce an entire scene by aligning all the acquired image sequences. A registration algorithm is necessary to tolerance as much as possible for intensity and geometric variation among images. However, captured image views of real scene usually produce unexpected distortions. They are generally derived from the optic characteristics of image sensors or caused by the specific scenes and objects.

**Methods and Findings:**

An analytic registration algorithm considering the deformation is proposed for scenic image applications in this study. After extracting important features by the wavelet-based edge correlation method, an analytic registration approach is then proposed to achieve deformable and accurate matching of point sets. Finally, the registration accuracy is further refined to obtain subpixel precision by a feature-based Levenberg-Marquardt (FLM) method. It converges evidently faster than most other methods because of its feature-based characteristic.

**Conclusions:**

We validate the performance of proposed method by testing with synthetic and real image sequences acquired by a hand-held digital still camera (DSC) and in comparison with an optical flow-based motion technique in terms of the squared sum of intensity differences (SSD) and correlation coefficient (CC). The results indicate that the proposed method is satisfactory in the registration accuracy and quality of DSC images.

## Introduction

Image registration is a fundamental technology in a variety of fields and has been extensively investigated over the past few decades. It has been applied to many areas, such as medical image analysis, surveillance operations, video representation and retrieval, remote sensing, and consumer device, with different registration techniques and performance requirements [1]–[8]. In 2D and 3D image registration, the state-of-the-art robust techniques are reviewed and discussed regarding their advantages, drawbacks, and practical implementations [9]. Image registration in similarity measure contains feature-based and intensity-based approaches. The former accounts for important features extracted from the image. The correspondence is then established between these features by measuring the similarity. The later compare intensities or other pixel-wise signatures directly without feature extraction. The feature-based registration is effective when distinctive image features exist, while the intensity-based registration is higher computational complexity. Several well-known examples of similarity functions are the sum of squared differences (SSD), cross-correlation (CC), mutual information (MI), normalized mutual information (NMI), and Markov-Gibbs random filed (MGRF)-based. The SSD and CC are common in image registration at the same modality, while the MI and NMI are suitable in multiple modalities. However, the MI and NMI do not account for spatial relationships between adjacent pixels and they are easily influenced by image noise. MGRF-based similarity measure is derived from an MGRF model of images. The image is used as a training sample to learn a characteristic structure of pairwise pixel dependencies and Gibbs potential functions of signal co-occurrences on these pairs [9]. Image registration is mainly the process of spatially registering acquired images so that corresponding features or pixels on them are consistent in geometry. A common registration problem for the application of consumer device is to align all the acquired image sequences into a complete scene. Image alignment requires a registration algorithm that will compensate as much as possible for geometric variability among images. However, captured image views of real scene usually produce different distortions. Some are derived from the optic characteristics of image sensors, and others are caused by the specific scenes and objects. In general, we would make some reasonable assumptions to develop a fast algorithm for real time applications in consumer device fields. That is, there are no moving objects in the scenes when capturing images, and the images are acquired in short time intervals.

Another important issue for image registration is to determine the transformation model. Depending on chosen type of spatial transformation, the required number of parameters for registration model would be decided. The rigid transformation model, which preserves relative distances of points, estimates the translation and rotation, whereas the affine model [10] estimates the rigid transformation parameters and the scale factor. The affine transformation preserves collinearity. That is, parallel lines are transformed into parallel lines, and the ratios of distances are preserved along parallel lines. In addition, a more complex transformation model, perspective projection [11], takes more parameters into account. It considers not only affine transformation but also the transformations of panning and tilting. The transformation model of perspective projection is estimated to apply to the images captured from a consumer device, such as a hand-held digital still camera and a CMOS image sensor.

Moreover, a registration algorithm usually minimizes a cost function that is a combination of an objective function and smoothness constraint [12]–[15]. There are various algorithms that iteratively minimize the surface distance in order to linearly align two regions, such as iterative closest point algorithms.

In this study, we propose an analytic approach to achieve deformable and robust point matching. A wavelet-based method is used to extract features and discard the noise in multiscale at the same time. It then speedily evaluates the spatial correspondence and geometrical transformation between two point sets with different sizes. It is robust to noise and tolerant to distortion caused by chromatic aberration and geometry discrepancy. Finally, a feature-based Levenberg-Marquardt algorithm (FLM) is used to further refine registration results and speedily obtain subpixel accuracy because of their feature-based characteristic.

The paper is organized as follows. Section 2 addresses the proposed method in detail. In Section 3, experimental results and a discussion for some validation examples are presented. Section 4 describes the limitations of present study and future works. Finally, the conclusion is given in Section 5.

## Methods

**Figure 1:**
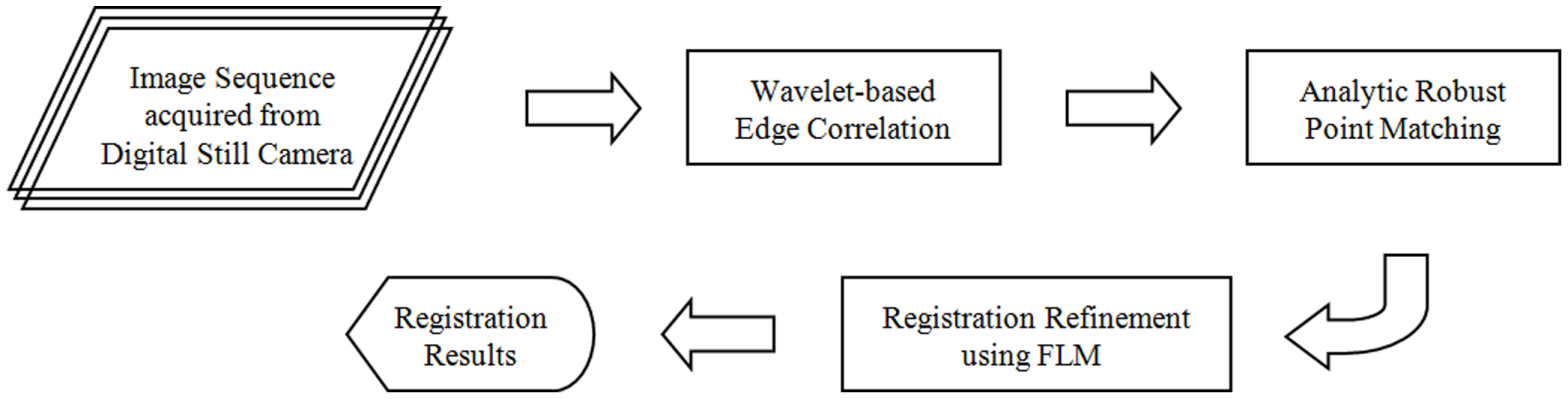
Flowchart of the proposed method. It consists of wavelet-based edge correlation, analytic robust point matching, and registration refinement with FLM.

The proposed method consists of feature extraction, image registration, and registration refinement. First, feature points are extracted by wavelet-based edge correlation with large responses in multiscale. An analytic robust point matching (ARPM) algorithm is then proposed to achieve deformable and accurate registration of feature point sets. Finally, a FLM method is proposed to further obtain subpixel accuracy. The flowchart of proposed method is illustrated in Fig. 1.

### 2.1. Feature Extraction

It is an important issue for feature-based image registration to extract significant features from acquired images, which will produce a great influence on the registration accuracy. More specifically, feature extraction is to extract representative features from the adjacent images, so as to effectively provide the geometrical and photometric information for image registration. Multi-resolution image decomposition is a useful technique for analyzing image information at various scales. Therefore, wavelet-based edge correlation, which had been verified the efficacy in feature extraction in our previous work [15], is used to extract the feature points with strong and consistent responses under different scales within a local area. In other words, feature points usually have larger values on the product of gradient moduli from multiscales, while the noise does not. In this study, Daubechies wavelet is used due to the special characteristic that it is compactly supported with extreme phase and highest number of vanishing moments for a given support width, which is beneficial to feature extraction [16].

**Figure 2:**
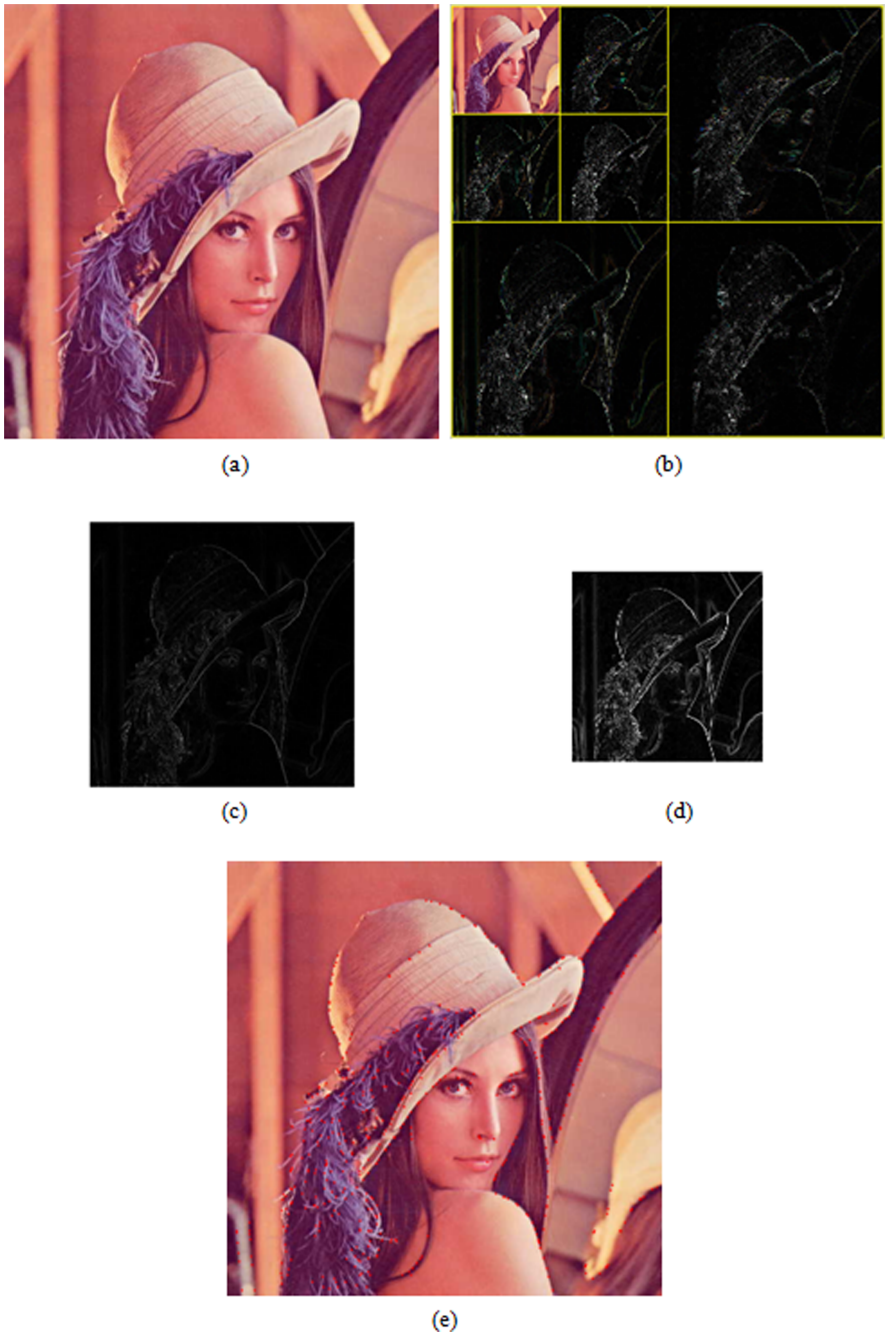
Procedure of feature extraction. (a) A test image, (b) 2D 2-level wavelet decomposition of test image (c), (d) gradient moduli (calculated from LH and HL quadrants) at level 1 and 2, respectively, (e) result of feature point extraction.

Due to the separable characteristic of wavelet transform, we represent 2D wavelet transform as two 1D ones in *x* and *y* directions, respectively,
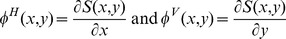
(1)where 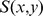
represents a 2D smoothing function. We denote 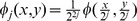 as a dilation function of 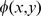 by a scaling factor *j*. The gradients 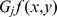 of an image 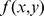 in the *x* and *y* directions and its modulus 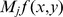 at level *j* are described as follows,
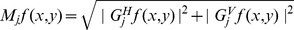
(2)where



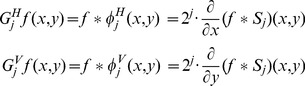
(3)All the edge points in image 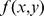 at level 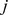 is located with local maxima of 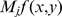. The edge correlation, which filters out the noise by a multiscale edge confirmation to detect reliable feature points, is represented as
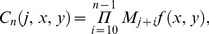
(4)where *n* is the level number, and *j* is the initial level. The true feature points can be obtained by means of edge correlation. Features and noise sometimes coexist in the wavelet domain, but features can usually exist in multiscales while noise can not [15]. In addition, the level number *n* is chosen as two to suppress the edge-bias problem of wavelets at multi-level. In this study, the observed property is used to distinguish the true feature points from noise. The procedure of feature extraction is shown in Fig. 2. A test image is given in Fig. 2(a). Fig. 2(b) shows 2D two-level wavelet decomposition for the test image. The gradient moduli of test image at level 1 and 2 are illustrated in Fig. 2(c) and Fig. 2(d), respectively. Finally, Fig. 2(e) shows the result of feature point extraction.

### 2.2. Image Registration

The RPM algorithm is a robust method for point-based registration [17]. Following the notation, we describe the proposed ARPM algorithm in detail.

Given two point-sets 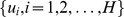 and 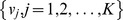 extracted from adjacent slices, and the correspondence mapping is denoted by a matrix *M* consisting of *m_ij_*. The entire energy function minimized by the ARPM algorithm is as follows:
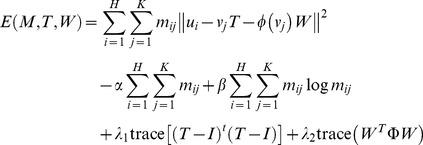
(5)where 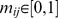 and it subjects to 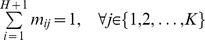 and 
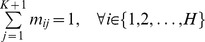
, *T* and *W* represent the geometric transformation and warping coefficient matrix respectively, and 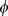 stands for the warping function. The size of matrix *M* is 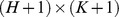 and its inner 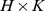 portion indicates the correspondence information for two point-sets. If a point *u_i_* corresponds to a point *v_j_*, then the entry *m_ij_* of the correspondence matrix *M* is equal to 1; otherwise, it is assigned to zero. In addition, in order to take the outliers into account so as to still hold the constraints of the row and column summation to one, an additional row and column is appended to the suffix of the correspondence matrix *M*.

All the components of the energy function are interpreted in turn in the following: The first term 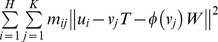 is the error term that it describes a corresponding problem by means of the perspective transformation. It is a desirable transformation since the rotation, scaling, translation, and global shear are all taken into account. The second term 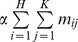 with the weighting 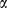 is used to avoid excessively much null correspondence. If 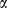 is large, then fewer points are discarded as outliers. The third term 
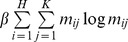
 with the temperature parameter 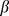 is an entropy function that it avoids the elements of correspondence matrix *M* being negatives. The fourth term 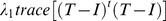 with the weighting 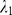 is a constraint on the geometric transformation *T* by means of the penalty on the remainder of subtracting the identity matrix *I* from the correspondence matrix *T*. The final term 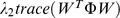 with the weighting 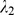 is a constraint on the warping coefficient matrix *W* by means of the penalty on the warping coefficient matrix *W*.

As above mentioned, the minimization problem in equation (5) mainly consists of two related sub-problems: the point-sets correspondence and the geometric transformation between two adjacent slices. Given the point-sets correspondence, the geometric transformation can be evaluated by resolving the constrained least-squares problem. Given the geometric transformation, the point-sets correspondence is found and achieved by resolving the linear assignment problem. Inspired by the idea, the algorithm incorporates the update scheme by alternating the update of the correspondence and the transformation parameters while keeping the other fixed, it is expected to jointly improve the two solutions as well as finally converge to the optimal solution.

The registration algorithm mainly consists of two principal steps. It is accomplished by using alternating update scheme. The first step is to update the point-sets correspondence matrix *M* as well as make sure that *M* corresponds to the row and column summation constraints all the time by keeping *T* and *W* fixed, with its currently evaluated transformation. Afterwards, the solution for correspondence matrix *M* could be calculated by means of the differentiation of the energy function in equation (5) with respect to *m_ij_*,
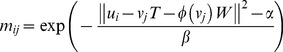
(6)


The second step is to update the parameters of geometric transformation *T* and warping coefficient matrix *W* with the correspondence matrix *M* held fixed. We propose an analytic differential approach, which is namely ARPM in this study, to evaluate the parameters of *T* and *W* by means of the differentiation of the energy function in equation (5) with respect to *T_pq_* and *W_pq_* respectively,
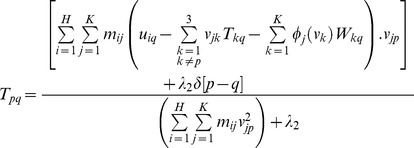
(7)

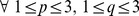


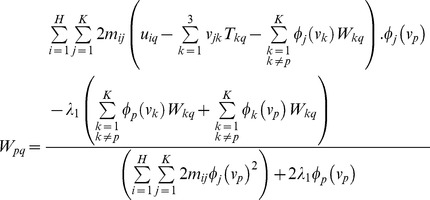


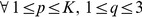
(8)where 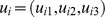, 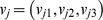, 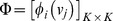, 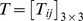, and 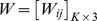. More specifically, *W_ij_* represents the element of matrix *W* that is located in the *p* ^th^ row and in the *q* ^th^ column. 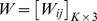 stands for the size of matrix *W* being *K* by 3. 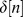, the unit sample sequence, is defined: 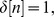 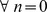; otherwise, 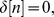 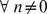. The two steps are iteratively performed while the temperature parameter 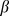 as well as the weighting 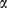 is gradually decreased. The decreasing process for the temperature parameter 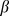 is similar to the deterministic annealing procedure [18]. The deterministic annealing with the temperature parameter 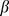 is a procedure to adjust the flexible degree of the correspondence matrix *M*. The correspondence matrix *M* eventually approaches a binary-values matrix as the temperature 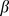 is gradually annealing. In addition, due to the property that deterministic annealing can escape from the local minima, the approach is guaranteed to obtain the near-optimal solution.

### 2.3. Registration Refinement

To make the registration result more accurate to further achieve subpixel precision, a refined step for image alignment is necessary. An FLM method, which is modified from [15], is proposed to apply to this study. Due to the feature-based characteristic of FLM method, it converges much faster than most other ones as long as the initial estimation is close to the global optimal solution. The sum of squared Euclidean distance errors for two feature-point sets is minimized to measure the similarity. The residue *E*, the sum of squared Euclidean distance errors, for the optimization criterion is written as
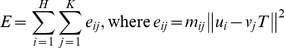
(9)where *H* and *K* stand for the point numbers of two point sets, which have been mentioned above. In order to resolve the nonlinear least-square minimization problem by the iterative FLM method, the Hessian matrix *A* and the gradient vector *b* must be calculated respectively,
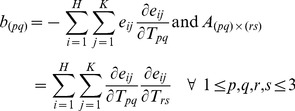
(10)where 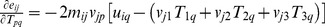. The parameter 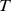 is then recursively updated,
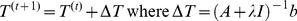
(11)where 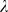 is a positive parameter, which is adjusted according to the convergence or divergence of the sum of squared errors. In other words, when the error sum decreases, the parameter 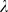 decreases and the next estimated update 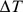 tends to the Newton method, while the parameter 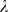 increases and the next estimated update 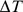 tends to the gradient descent approach if the error sum increases. In this study, 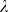 is initialized as 1. The FLM method is performed repeatedly until either the relative error 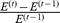 is less than a threshold or predefined steps are reached.

## Experimental Results and Discussion

### 3.1. Several Examples of the Registration

**Figure 3:**
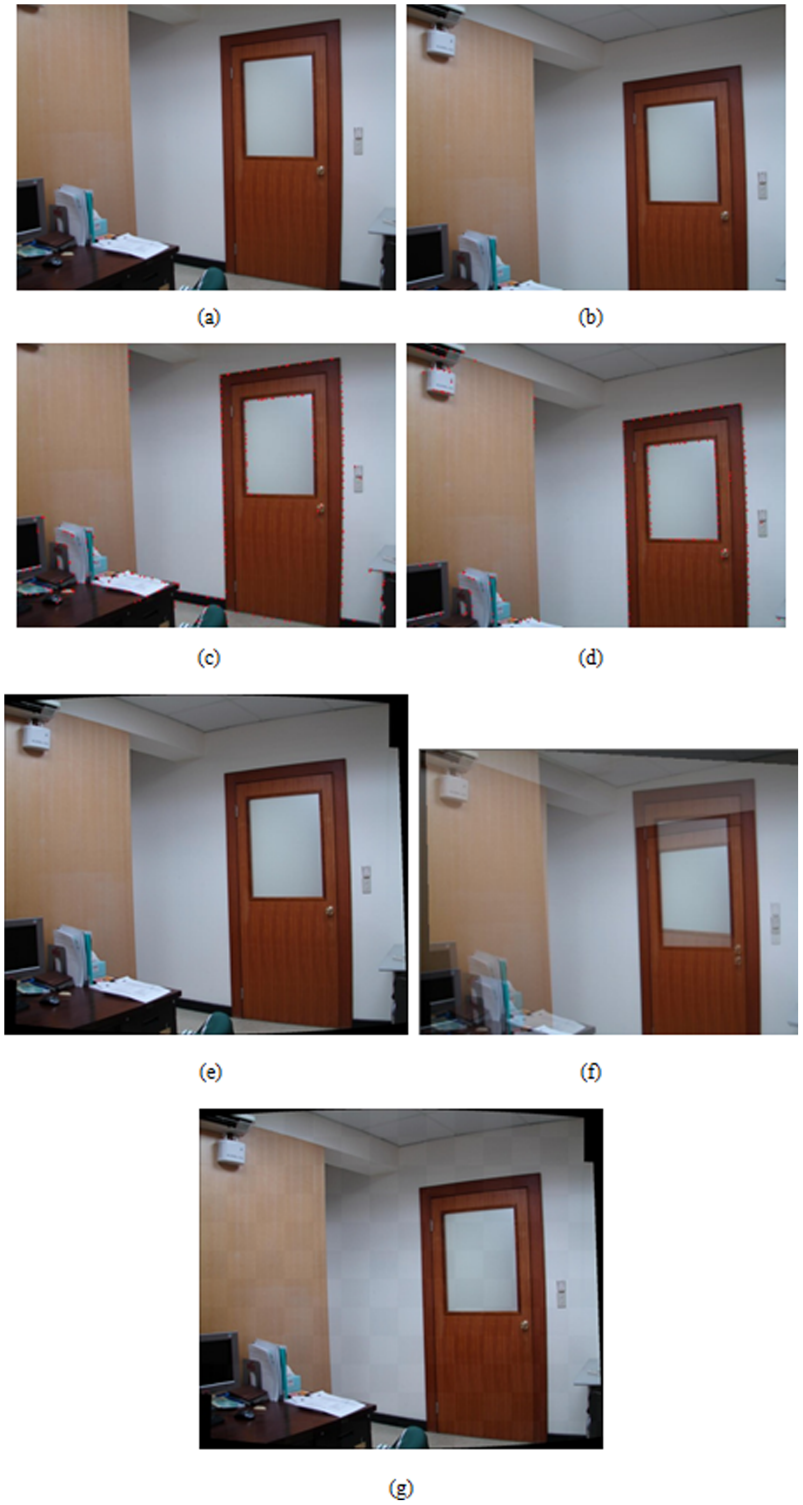
Registration result of indoor desk and door. (a), (b) An image pair used for registration, (c), (d) results of feature point extraction from (a) and (b), respectively, (e) registered result with proposed algorithm, (f) registered result by optical flow-based approach, (g) checkerboard visualization after the registration by proposed algorithm.

**Figure 4:**
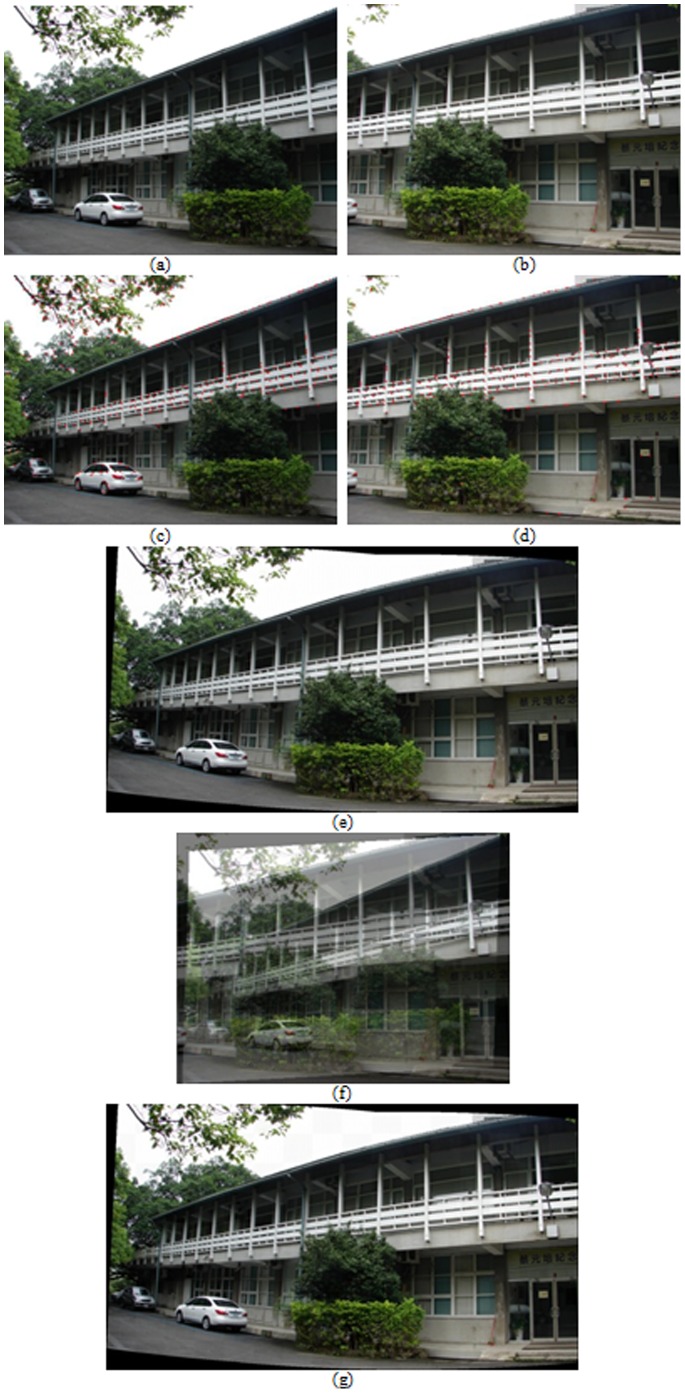
Registration result of building at a near distance. (a), (b) An image pair used for registration, (c), (d) results of feature point extraction from (a) and (b), respectively, (e) registered result with proposed algorithm, (f) registered result by optical flow-based approach, (g) checkerboard visualization after the registration by proposed algorithm.

**Figure 5:**
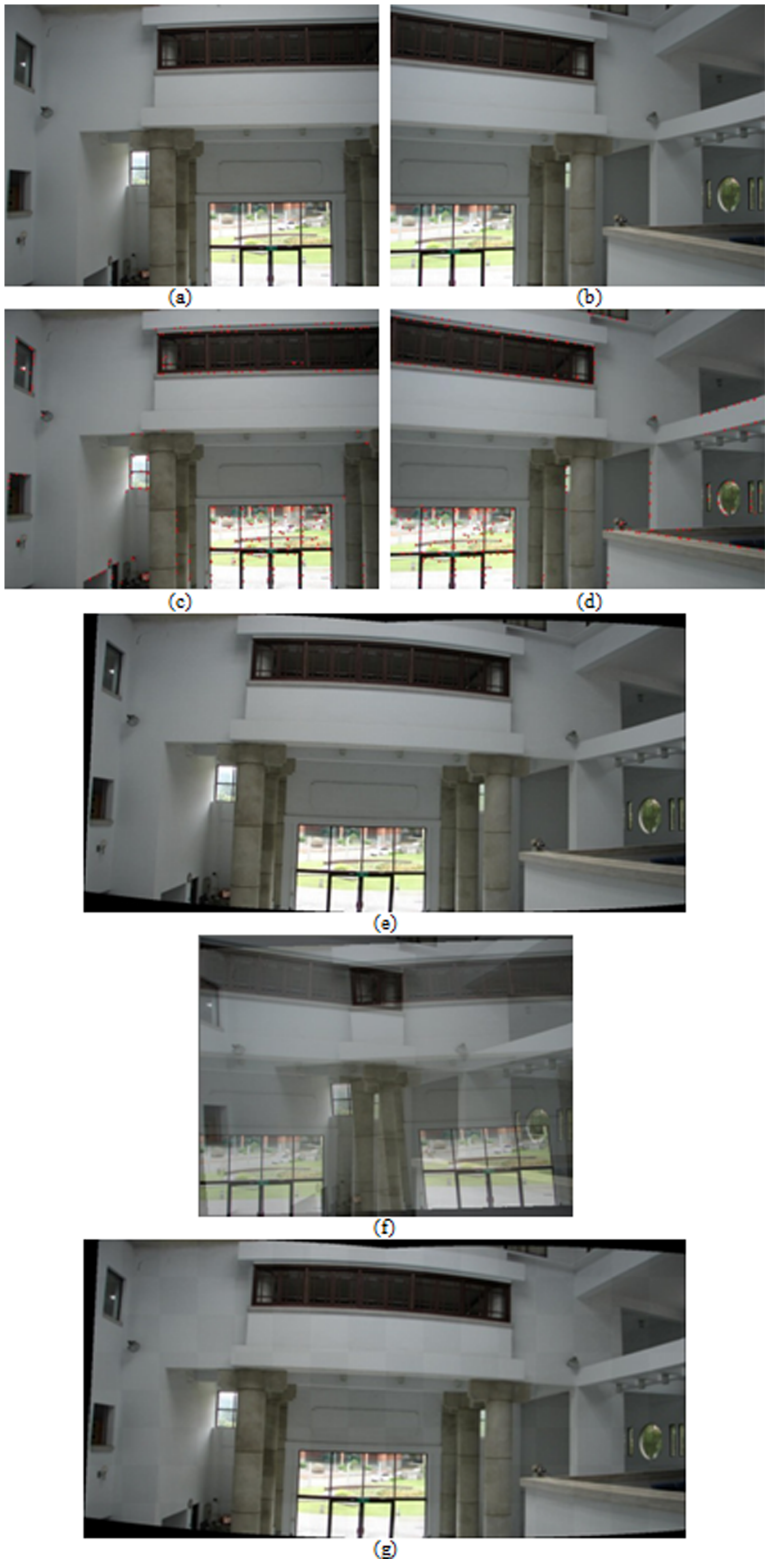
Registration result of building indoors. (a), (b) An image pair used for registration, (c), (d) results of feature point extraction from (a) and (b), respectively, (e) registered result with proposed algorithm, (f) registered result by optical flow-based approach, (g) checkerboard visualization after the registration by proposed algorithm.

A hand-held digital still camera is used to capture images in the experiments. Each image is obtained with the resolution of 1024×768 pixels in 24bit RGB format. A wide set of real image sequences are acquired and tested to evaluate the performance of proposed algorithm by means of the visual quality assessment. The feature extraction and registration results for a variety of image pairs are shown in Fig. 3, 4, and 5. More specifically, subfigures (a) and (b) of each figure show the image pair that will be used for registration. The results of feature point extraction from subfigures (a) and (b) are shown in subfigures (c) and (d), respectively. Finally, the registration result of image pair with proposed algorithm is shown in subfigure (e). The geometric transformation *T*s of these three image pairs from Fig. 3, 4, and 5 are listed in Table 1. In addition, an optical flow-based motion algorithm [19] is implemented for comparison. The algorithm is a registration technique that takes motion estimation into account. It produces the optical flow field, a collection of two-dimensional velocity vectors, one for each small region of the image [19]. The registered results of this compared algorithm for the same image pairs are shown in Fig. 3(f)-5(f). In addition, the checkerboard visualization is also used to visualize the registration results, as shown in Fig. 3(g)-5(g), of the proposed method. The visual demonstrations indicate that the proposed method achieves better and finer results than the compared approach.

**Table 1:** Geometric transformation *T*s of three image pairs (IP) from Fig. 3–5.

*T*	*T_11_*	*T_12_*	*T_13_*	*T_21_*	*T_22_*	*T_23_*	*T_31_*	*T_32_*	*T_33_*
IP 1 (Fig. 3)	0.996	0.001	−73.5	−0.007	1.003	−29.4	0.089	0.031	0.997
IP 2 (Fig. 4)	0.997	−0.056	−1.8	0.058	0.968	267.8	−0.020	−0.264	0.961
IP 3 (Fig. 5)	0.998	−0.049	−11.7	0.054	0.931	380.1	−0.005	−0.374	0.926

The setup of parameters in this experiment for the ARPM algorithm is described in detail as follows. The initial value for the temperature 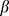 is assigned to slightly more than the longest distance of all point pairs, and it then gradually decreases with the annealing rate 0.93. The weighting 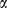 is assigned to 5, whereas the weightings 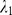
and 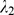 are set to 0.1 and 0.5, respectively. The point-sets correspondence *M* is initialized such that all the inner entries are 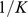 and the outlier ones are 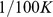. The geometric transformation *T* is initialized to a unit matrix. It is generally sufficient to achieve converged results by the alternating update on the correspondence *M* and geometric matrix *T* 20 runs.

Fig. 3 shows the image panning and tilting problems for indoor desk and door. To show the importance of proposed registration algorithm, the image pair is captured with a large vertical motion to make big difference. Fig. 3(e) is the result of proposed registration algorithm, whereas Fig. 3(f) is the result of optical flow-based approach. As seen in these two images, it can reveal that it is difficult for optical flow-based registration approach to handle large displacement problems. The results indicate that the proposed algorithm can resolve the panning and tilting problems to achieve accurate registration, while the optical flow-based approach cannot.

Fig. 4 shows the registered results of image pairs acquired from the buildings with near distances. The displacements between the image pair are quite large in this case, so the optical flow-based approach cannot obtain precise registration. The results shown in Fig. 4(e) indicate that the proposed algorithm concerns large displacements of image pairs and distortions produced from the perspective projection of acquired images.

Fig. 5 shows the results of registration for image pairs with large perspective distortion in panning direction. The optical flow-based approach cannot register well since the perspective distortion is too large to accurately calculate the motion flow from matching points of image pairs. Fig. 5(e) reveals that the proposed algorithm can achieve satisfactory registration results even if the distortion in perspective projection is considerably large in titling and panning directions.

### 3.2. Quality of the Registration

The proposed registration algorithm has been applied to all the image pairs. To assess the quality of the registration, we calculate the mean and standard deviation of the squared sum of intensity differences (SSD) as well as the correlation coefficient (CC).
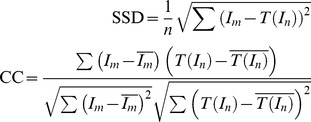
(12)where *I_m_* and *I_n_* represent a pair of images. *T*(.) is the geometric transform evaluated after each registration step. 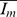 and 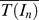 denote the average intensities. *n* is the pixel number within the overlapping zone. For each image sequence, the SSD and CC provide an indirect measure of registration quality.

It is expected in theory that the image difference only shows the underlying noise from image acquisition. However, the effects of the misregistration, geometric deformation are clearly visible in registered images. In the experiments, we compare the proposed registration method with the optical flow-based motion algorithm [19]. Table 2 and 3 summarize the average results of registration quality in terms of SSD and CC for these two registration algorithms for synthetic and real DSC images, respectively. Table 2 lists the registration results for the synthetic images, which are selected from 20 DSC images with randomly selected parameters of geometric transform and Gaussian noise. The parameters for geometric transform *T* are selected in the ranges of 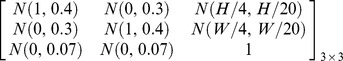, where *H* and *W* represent the height and width of images, respectively; the parameters for Gaussian noise are selected in the ranges of *N*(0, 5
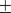
2). Table 3 lists the registration results evaluated from all the pairs of DSC images. The results indicate that the proposed algorithm can achieve satisfactory registration accuracy and quality, which is better than the optical flow-based approach.

**Table 2:** Comparison of average registration quality from several (20) synthetic DSC images in terms of SSD and CC for two registration algorithms.

Registration Quality for Synthetic Images	SSD (mean 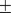 standard deviation)	CC
Optical Flow-based Motion Approach	74.93 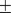 9.54	0.559
Proposed Algorithm	11.36 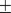 2.95	0.903

**Table 3:** Comparison of average registration quality from all the pairs of DSC images in terms of SSD and CC for two registration algorithms.

Registration Quality for Real Images	SSD (mean 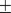 standard deviation)	CC
Optical Flow-based Motion Approach	142.17 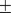 17.65	0.268
Proposed Algorithm	9.70 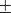 2.08	0.931

### 3.3. Statistical Evaluation

To validate whether these two algorithms are significantly different or not, one-way analysis of variance (ANOVA) is performed for the analysis of SSD and CC on both the synthetic and real data. The statistical analyses with one-way ANOVA are used to evaluate if the difference is significant for the factor SSD or CC.

We obtain *p*-values less than 0.0001 and less than 0.0001 for SSD and CC respectively for synthetic data, while the *p*-values are less than 0.0001 and less than 0.0001 for SSD and CC respectively for real data. The results of test indicate that these two algorithms are significantly different among them. More detailed comparisons of *p*-values between them are then performed. The results indicate that there are significant differences in the estimation of SSD and CC between the optical flow-based approach and proposed algorithm for synthetic data (*p*-values be <0.0001 and <0.0001 for SSD and CC, respectively). In addition to synthetic data, the results of tests for real data are also discussed. The results show that the proposed algorithm is significantly better than optical flow-based approach in SSD and CC estimation (*p*-values be <0.0001 and <0.0001 for SSD and CC, respectively). Accordingly, the proposed algorithm obtains promising performance in the evaluation of registration quality for both the synthetic and real data.

### 3.4. Computational Cost

In addition, the computational cost is also considered to evaluate the efficiency of the proposed algorithm in this study. Image pairs are registered on a PC with an Intel Core 2 Duo E6300 processor and 1GB RAM. The results show that the proposed algorithm takes average 3.74±0.25 (mean ± standard deviation) seconds for image pairs acquired from Fig. 3, 4, and 5, whereas the optical flow-based approach takes average 5.08±0.21 seconds. It indicates that the proposed method is less time consuming in computational cost.

### Limitations of Present Study and Future Works

Although the proposed algorithm has less computational cost, it doesn’t achieve the performance in on-line applications. In future works, the algorithm will be modified to overcome it as much as possible. In addition, the algorithm will be also applied to other style image sequences, such as medical images and satellite/remote sensing images.

### Conclusion

In this study, we have presented an analytic registration algorithm for the applications of scenic images. The multi-scale concept is exploited to retain significant features and discard the noise by wavelet transform. An analytic approach is then proposed to achieve deformable and accurate registration. Finally, we refine registration accuracy to subpixel precision by the FLM method. It reduces the computational cost quite significantly due to its feature-based characteristic. It shows that this study is fairly valuable for equipping the consumers with a powerful tool in life applications.
